# Efficacy and safety of adenosine for supraventricular tachycardia: A meta-analysis utilizing BioMedGPT-LM-7B

**DOI:** 10.1186/s12872-025-04595-x

**Published:** 2025-03-07

**Authors:** Xuemei Feng, Jia Liu

**Affiliations:** 1 School of Basic Medical Sciences, Shanghai Jiaotong University, Shanghai, 200025 China; 2 School of International Pharmaceutical Busines, China Pharmaceutical University, #639 Longmian Dadao, Jiangning District, Nanjing, 211198 China

**Keywords:** Adenosine, Calcium channel blockers, Supraventricular tachycardia, Meta-analysis, Conversion to sinus rhythm, BioMedGPT-LM-7B

## Abstract

**Background:**

Patients with supraventricular tachycardia (SVT) often experience multiple clinical symptoms that require emergency treatment. This study utilized BioMedGPT-LM-7B, an artificial intelligence (AI) model, to comprehensively evaluate the efficacy and adverse effects of adenosine/adenosine triphosphate (ATP) versus calcium channel blockers (CCBs) in SVT treatment.

**Methods:**

This study conducted a comprehensive search of multiple medical databases, as well as major trial registries up to December 2024. We performed dual screening and assessment using BioMedGPT-LM-7B and the traditional Cochrane bias risk tool. The primary outcomes were the rate of sinus rhythm restoration and major adverse events, while secondary outcomes included time to restoration, relapse to SVT post-reversion, and any minor adverse events. Outcome measurements were based on odds ratios (OR) and Mean Difference (MD), with the quality of primary outcomes assessed using the GRADE method.

**Results:**

This study included 10 RCTs with a total of 960 SVT patients admitted to the emergency department. Comparing BioMedGPT-LM-7B with the traditional Cochrane bias risk tool, we found no significant differences in random sequence generation and selective reporting. Moderate evidence showed no difference between adenosine/ATP and CCBs in restoring sinus rhythm (OR = 1.44, 95% CI [0.89,2.34]), but adenosine/ATP had a shorter time to reversion (MD = 423,24, 95% CI [293.54, 552.93]). However, the research findings show a lower level of evidence regarding differences in side effects among the drugs mentioned above. Three cases of hypotension were reported in the CCB group, whereas none were reported in the adenosine group.

**Conclusion:**

Adenosine/ATP and CCBs have similar efficacy in treating SVT, but adenosine/ATP has a shorter conversion time and no reported cases of hypotension. Clinical studies indicate that adenosine has a higher success rate and faster conversion time in restoring sinus rhythm compared to ATP, with milder side effects. However, further prospective studies are needed to evaluate patient experience and potential adverse events, ensuring a more comprehensive understanding of treatment safety and efficacy. Additionally, this study showcases BioMedGPT-LM-7B’s potential for medical data analysis and future meta-analyses.

**Supplementary Information:**

The online version contains supplementary material available at 10.1186/s12872-025-04595-x.

## Introduction

Supraventricular tachycardia (SVT) is a arrhythmia originating from the supraventricular tissues of the heart. It is more commonly observed in women, with an average onset age of approximately 55 years, impacting patients’ quality of life. Treatment options are diverse and include vagal maneuvers, adenosine/adenosine triphosphate (ATP), calcium channel blockers (CCBs), electrical cardioversion, etc [1, 2]. Adenosine/ATP terminates paroxysmal supraventricular tachycardia (PSVT) in acute treatment by inhibiting atrioventricular (AV) node conduction, with rapid and transient effects and very short half-lives. Adenosine blocks calcium influx mediated by cAMP and increases potassium conduction, which rapidly suppresses AV node conduction, potentially aiding in the temporary restoration of sinus rhythm. ATP is rapidly metabolized to adenosine in the body, and its action depends on this conversion. Adenosine has milder side effects, typically presenting as mild discomfort such as facial flushing. In contrast, ATP is associated with more significant side effects, including bradycardia, AV block, or cardiac arrest [3, 4]. On the other hand, CCBs are also used to treat SVT. They inhibit voltage-dependent calcium channels, reduce intracellular calcium levels, and block calcium-dependent conduction through the atrioventricular node. However, the use of CCBs can result in negative inotropic effects and peripheral vasodilation, requiring caution, particularly in patients with compromised left ventricular function [5]. Although adenosine/ATP and CCBs have been shown to effectively treat SVT, some clinical trials indicate that their relative efficacy and safety profiles differ [6, 7]. Thus, a meta-analysis is necessary to clarify these issues.

In recent years, artificial intelligence (AI), particularly in natural language processing, has made significant strides, with generative language models like OpenAI’s ChatGPT excelling in various linguistic tasks and successfully applied in the medical field [8–10]. BioMedGPT-LM-7B, developed by the Tsinghua team, is an AI model trained on medical texts using Llama2, demonstrating strong natural language processing capabilities in medical contexts [11]. Although generative language models have been successfully applied in areas such as health management, personalized treatment planning, and assisting clinical decision-making [8–9], their application in clinical research is still in the exploratory stage. This study aims to evaluate using the BioMedGPT-LM-7B model in evaluating the efficacy and safety of adenosine/ATP and CCBs in the treatment of SVT through systematic reviews and meta-analyses of randomized controlled trials (RCTs). The research seeks to provide stronger evidence for clinical decision-making and further advance the application of generative language models in the medical field.

## Methods

### Study design

First, RCTs that met the standards for randomization and treatment allocation were included, excluding studies with protocol violations or unclear randomization [12, 13]. The study population consisted of patients of any age diagnosed with SVT within 24 h using a 12-lead electrocardiogram (ECG), excluding those with electrophysiologically induced SVT in the laboratory. Eligible studies had to compare intravenous CCBs (such as verapamil and diltiazem) with intravenous adenosine/ATP, regardless of dose or infusion rate. After obtaining eligible RCTs, we parsed and analyzed the data using BioMedGPT-LM-7B, including data extraction and quality assessment. The meta-analysis was performed with RevMan 5.3 software, and we evaluated the quality of evidence for each prespecified outcome according to the Grading of Recommendations, Assessment, Development, and Evaluations (GRADE) method. The rigor of the study and the scientific validity of the results could be enhanced through the detailed experimental design and standardized experimental process.

### Outcome measures

This research aimed to identify the rate of sinus rhythm restoration and the occurrence of clinically relevant adverse events. In addition to these primary outcomes, the study also examined secondary outcomes, which included the time to restore sinus rhythm, the incidence of recurrence of SVT within two hours after restoration, and minor adverse events.

### Search and selection strategies

We integrated the natural language processing capabilities of BioMedGPT-LM-7B. First, we used BioMedGPT-LM-7B to generate a series of search strategies related to RCTs by repeatedly inputting terms such as “adenosine/ATP” and “supraventricular tachycardia” until two experts reached an agreement. Second, as of December 2024, this study used BioMedGPT-LM-7B to generate search formulas for conducting a broad search of RCTs related to “(adenosine OR adenosine triphosphate OR ATP) AND (supraventricular arrhythmia OR SVT OR PSVT).” Our research involved systematic exploration across multiple databases, including the Cochrane Central Register of Controlled Trials (CENTRAL), China National Knowledge Infrastructure (CNKI), Epub Ahead of Print, In-Process & Other Non-Indexed Citations of MEDLINE and Embase. For increased sensitivity, the Cochrane RCT filter was utilized during our MEDLINE exploration, and terms advised by the Cochrane Handbook for Systematic Reviews of Interventions were employed in our search through Embase [14]. There were no limitations on the search results by language or publication date.

### Data extraction and collection

After a comprehensive search, two reviewers independently screened titles and abstracts to identify relevant studies, categorizing them as “retrieve” or “not retrieve.” Discrepancies were resolved by a third reviewer. Full texts of eligible studies were retrieved and independently assessed by two reviewers for inclusion or exclusion, with reasons documented. Duplicate records were removed to ensure each study’s unique identity was preserved. Retrospective, observational, and review studies that did not meet the inclusion criteria were excluded. To maintain consistency, data extraction was conducted using a standardized form. Detailed data on the included studies, participants, interventions, and outcome measurements were extracted and recorded.

### Data preprocessing and analysis

Two reviewers independently assessed the risk of bias in the included studies, following the criteria outlined in the Cochrane Handbook for Systematic Reviews of Interventions [14]. This included: the method of generating randomized sequences, concealment of allocation, blinding of patients and trial personnel, blinding of outcome assessors, completeness of outcome data, selective outcome reporting, and other potential sources of bias. These criteria were graded as posing a high, low, or unclear risk of bias. Discrepancies were resolved through discussion, and further clarification was sought from study authors when necessary.

**Fig. 1:**
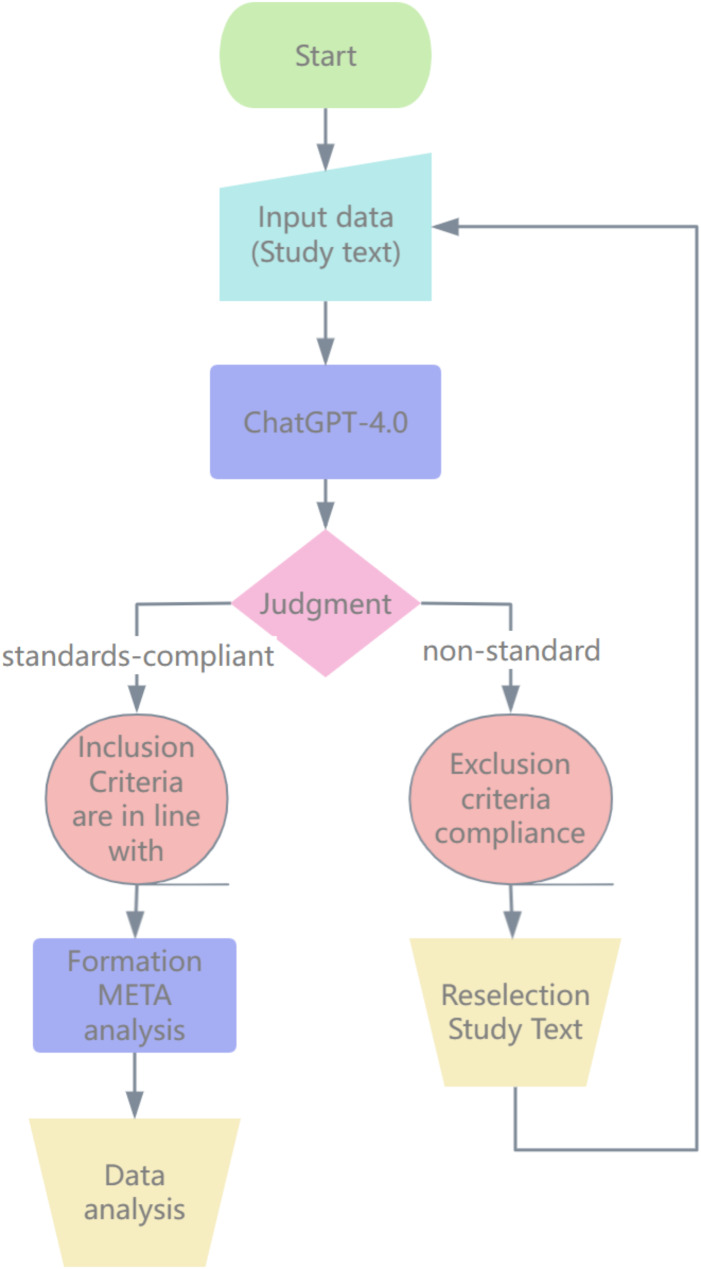
Risk of bias for trained BioMedGPT-LM-7B

Subsequently, we integrated BioMedGPT-LM-7B with the Cochrane Risk of Bias Tool for an effective evaluation of bias risk within the studies. First, we trained BioMedGPT-LM-7B with a set of RCTs that had already been assessed by the Cochrane Risk of Bias Tool, providing inputs that included the study text and the corresponding bias assessment results. From this, BioMedGPT-LM-7B learned the relationship between textual features and bias ratings, e.g., how to determine the bias of the randomization process from the description of its risk. Then, we entered new RCTs into BioMedGPT-LM-7B, which can automatically assess the risk of bias rank of these studies based on previous findings (Fig. 1).

Data analysis was performed utilizing RevMan 5.3 software. For continuous outcomes, we calculated mean differences (MDs) alongside 95% confidence intervals (CIs), and for dichotomous outcomes, we computed odds ratios (ORs) with 95% CIs. Analysis was based on individual participant data, using only the pre-crossover phase due to insufficient drug washout periods. Heterogeneity between studies was assessed using chi-square tests and the I² statistic. A fixed-effects model was applied if *p >* 0.10; otherwise, a random-effects model was employed. I² values were categorized as low (0–25%), moderate (25–50%), or substantial (50–100%). Funnel plots were planned to assess publication bias for outcomes with more than ten studies, but could not be performed due to the insufficient number of studies [14]. Subgroup analyses were planned based on demographic factors (e.g., age, sex, comorbidities) to explore their effects on treatment outcomes. However, subgroup analyses for demographic factors could not be conducted due to insufficient data. Sensitivity analyses by excluding high-bias studies were also not performed, as all included studies had at least one high-bias risk.

### Comparative experiment

In designing the comparative experiment, our goal was to evaluate and compare the performance of experts, GPT-4.0, standard Llama2, and BioMedGPT-LM-7B in handling medical research data. The key assessment metrics included accuracy, speed, data handling capacity, scope of expertise, automation capability, and risk of bias. By setting a series of specific tasks and challenges, these tasks aimed to simulate the data processing needs in real-world medical research, including data cleaning, data analysis, interpretation of results, and literature review, among others.

#### Accuracy

##### Definition

Accuracy was defined as the proportion of correctly identified outputs compared to the known ground truth in the dataset.

##### Implementation

The same standardized medical dataset, containing tasks such as identifying specific patterns, errors, and outliers, was assigned to each participant (both human and AI models). The accuracy of outputs was calculated by comparing the results with the ground truth, as shown in Eq. (1).

1 $$\,\text{Accuracy\,}\left(\%\right)=\frac{\text{EquationNumber\,of\,correct\,outputs}}{\text{total\,EquationNumber\,of\,outputs}}\times\,100\%$$

#### Speed and data handling capacity

##### Speed definition

Speed was calculated as the number of data entries processed per hour.

##### Speed implementation

A fixed number of data entries (e.g., 10,000) were assigned to each participant, and the time taken to complete the task was recorded, as shown in Eq. (2).

2 $$\text{Speed\,(entries/hour)=}\frac{\text{number\,of\,processed\,entries}}{\text{time\,(in\,hours)}}$$

##### Data handling capacity definition

The maximum number of data points that could be processed without performance degradation.

##### Data handling capacity implementation

Participants were tasked with handling increasingly larger datasets until their performance, defined by accuracy or time efficiency, dropped below 90% of the initial baseline.

#### Scope of expertise

##### Definition

The breadth of applicability within the medical field was assessed by assigning tasks across diverse medical subdisciplines, including cardiology, pharmacology, epidemiology, and biostatistics.

##### Scope of expertise scoring

Each task was evaluated as correct or incorrect based on predefined criteria, as shown in Eq. (3).

3 $$\text{Scope\,of\,}\text{e}\text{xpertise\,=}\frac{\text{relevant\,applications}}{\text{total\,applications}}$$

#### Automation capability

##### Definition

Automation capabilities were defined as the degree of manual intervention required during task execution.

##### Implementation

The number of manual interventions needed per task was recorded as an objective measure, with fewer interventions indicating a higher level of automation capability. This metric aimed to quantify the autonomy of each system in completing assigned tasks.

#### Risk of bias

##### Definition

Bias risk was evaluated by examining systematic deviations or patterns of bias in the outputs.

##### Implementation

Each output was reviewed by three independent experts, who assigned an average bias score based on the observed trends. Bias was categorized into low, moderate, or high levels, depending on the degree of deviation from the ground truth and cross-validation results from the review team.

#### Experiment control and validation

To ensure uniformity and reliability across participants, each metric was tested under controlled conditions. Tasks were repeated three times, and the average results were recorded to address variability. A dedicated evaluation team independently validated the performance outcomes of all participants, including experts, GPT-4.0, Llama2, and BioMedGPT-LM-7B, ensuring the robustness and reproducibility of the comparative analysis.

## Results

### Included studies and participants

**Fig. 2:**
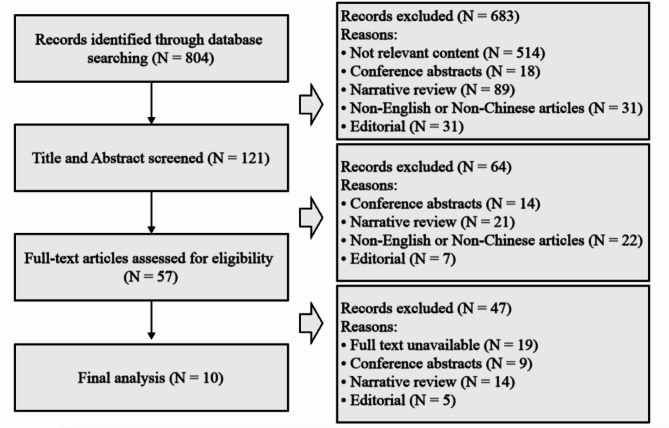
Research flowchart

In our updated literature review, we identified 804 new references. After removing duplicates, 121 records were screened by titles and abstracts. Most were excluded for not being RCTs or lacking a comparison between adenosine/ATP and CCB. Despite thorough reference checking, no additional trials were found. The original review included 10 trials published between 1982 and 2013 [15–24] (Fig. 2), involving 960 participants (Table 1). Only one trial included participants above the age of 10, while all others enrolled only adults [15]. Although the number of participants under 18 in this trial is unclear [16], all studies included patients with SVT.

**Table 1:** Characteristics of included studies

Inclusion of studies	Participants	Interventions	Outcomes
**Cabrera-Sole 1989**	Age not stated, presumed adult Gp 1: 44 participants Gp 2: 43 participants	Gp 1: ATP 20 mg bolus Gp 2: Verapamil 10 mg bolus	Reversion rate Minor A/E
**Cheng 2003**	Adults 18 to 75 years Gp 1: 60 participants (29 M) Gp 2: 62 participants (25 M)	Gp 1: Adenosine 3 mg, then 6 mg, then 9 mg every 1 to 2 min if no response to the previous dose. Mean dose 9.63 mg Gp 2: Verapamil 5 mg over 5 min, repeated if no reversion by 15 min. The mean dose of 7.15 mg	Reversion rate Time to reversion Minor A/E
**Ferreira 1996**	Adults Gp 1: 25 (8 M) Gp 2: 25 (9 M)	Gp 1: ATP 10 mg, then 20 mg bolus if needed. Mean dose 10.8 mg Gp 2: Verapamil infused at 5 mg/min up to 15 mg if needed. The mean dose of 9.38 mg	Reversion rate Time to reversion Recurrence rate Minor A/E Major A/E
**Gil Madre 1995**	Adults (25 M,25 F) Gp 1: 26 participants Gp 2: 24 participants	Gp 1: ATP 5 mg, then 10 mg, then 20 mg every 1 min if the previous dose is not effective Gp 2: 5 mg over 3 min, repeated after 10 min if no response to the first dose	Reversion rate Relapse rate Minor A/E
**Greco 1982**	Children < 13 years Gp 1: 20 participants Gp 2: 23 participants	Gp 1: ATP titrated to effect, mean dose 7.46 mg Gp 2: Verapamil titrated to effect, mean dose 2.09 mg	Reversion rate Minor A/E
**Lim 2009**	Adults Gp 1: 104 participants on adenosine, mean age 50.6 ± 17.0, 42% males Gp 1: 102 participants on verapamil (57 people) and diltiazem (59 people). Mean age 48.9 ± 18.3, 40% males	Gp 1: Adenosine, initially a 6-mg bolus, then a 12-mg bolus after 2 min, if needed Gp 2: Verapamil and diltiazem Verapamil: slow intravenous infusion at a rate of 1 mg per minute, up to a maximum dose of 20 mg Diltiazem: slow intravenous infusion at a rate of 2.5 mg per minute, up to a maximum dose of 50 mg Refractory cases were crossed over if the initial intervention was not successful after repeated admissions. These cases were counted as failures of the intervention and were not included in the final analysis.	Reversion rate Relapse rate: recurrences during 2-hour observation period Major adverse event: hypotension
**Vranic 2006**	Adults The mean age of men was 47 ± 12 years, and women 48 ± 12 years	Gp 1: Adenosine IV bolus of 6 mg, then 12 mg if needed Gp 2: Verapamil IV 5 mg up to maximum dose of 10 mg if needed	Cardioversion into sinus rhythm Duration to sinus rhythm conversion Relapse Biomarkers outcomes
**Ma 2011**	Adults Male: Female 14:13 Gp 1: 27 cases, age 42 ± 3 Gp 2: 27 cases, age 44 ± 2 Gp 3: 27 cases, age 43 ± 3	Gp 1: ATP: 10 ~ 15 mg direct rapid injection ( 1 ~ 2 s completion), then saline rapid rinse, no response within 3 ~ 5 min again 15 mg injection, the total amount of not more than 45 mg Gp 2: Propafenone: group with propafenone 70 mg diluted by 0.9% saline 20mL slowly static injection (5 ~ 10 min to complete), if there is no response, 10 ~ 20 min after the ineffective repeat static injection of 70 mg, the total amount of not more than 280 mg Gp 3: Verapamil: 5 mg added to 5% dextrose injection 20mL slow intravenous injection (time of about 5 ~ 10 min), if not effective, 15 ~ 20 min after repeated injection 5 ~ 10 mg	Reversion rate Time from dosing to termination of SVT Adverse effects: chest tightness, hypotension
Li 2005	Adults (18–72 years Gp 1: 25 cases, age (46.5 ± 14.5) years, male to female ratio: 12:13. Gp 2: 26 cases, age (49.2 ± 16.3) years, male to female ratio: 13:13.	Gp 1: Adenosine: rapid intravenous injection within 2 s, followed by rapid washout with saline. The initial dose is 3 mg, the 2nd dose is 6 mg, and the 3rd dose is 12 mg at 1 min to 2 min intervals, and the dose should not be increased if a high degree of atrioventricular block is present. Gp 2: Verapamil: 5 mg diluted and given intravenously for 5 min, if the seizure is not terminated, a further 5 mg can be given 15 min later at a rate of 1 mg/min, stopping immediately when the supraventricular tachycardia is terminated during the infusion.	Reversion rate Relapse Time to reversion Adverse effects: Low blood pressure, chest tightness, shortness of breath.
Wang 2013	Adults Gp 1: 103 cases, age (44.3 ± 5.1) years, male to female ratio: 35:68. Gp 2: 103 cases, age (44.1 ± 5.4) years, male to female ratio: 34:69.	Gp 1 = Adenosine: Initial dose: 6 mg intravenous bolus. If SVT is not terminated after 1–2 min, administer a second dose of 12 mg via slow intravenous bolus. If the tachycardia persists, repeat with the same doses and method up to 3 times. Gp 2 = Verapamil = 5 mg, diluted with 10 ml of 0.9% sodium chloride, and slowly injected intravenously over at least 2 min. If the tachycardia is not terminated, administer 0.15 mg/kg in 100–200 ml of 0.9% sodium chloride via intravenous drip for at least 1 h.	Reversion rate

### Risk of bias assessment

**Fig. 3:**
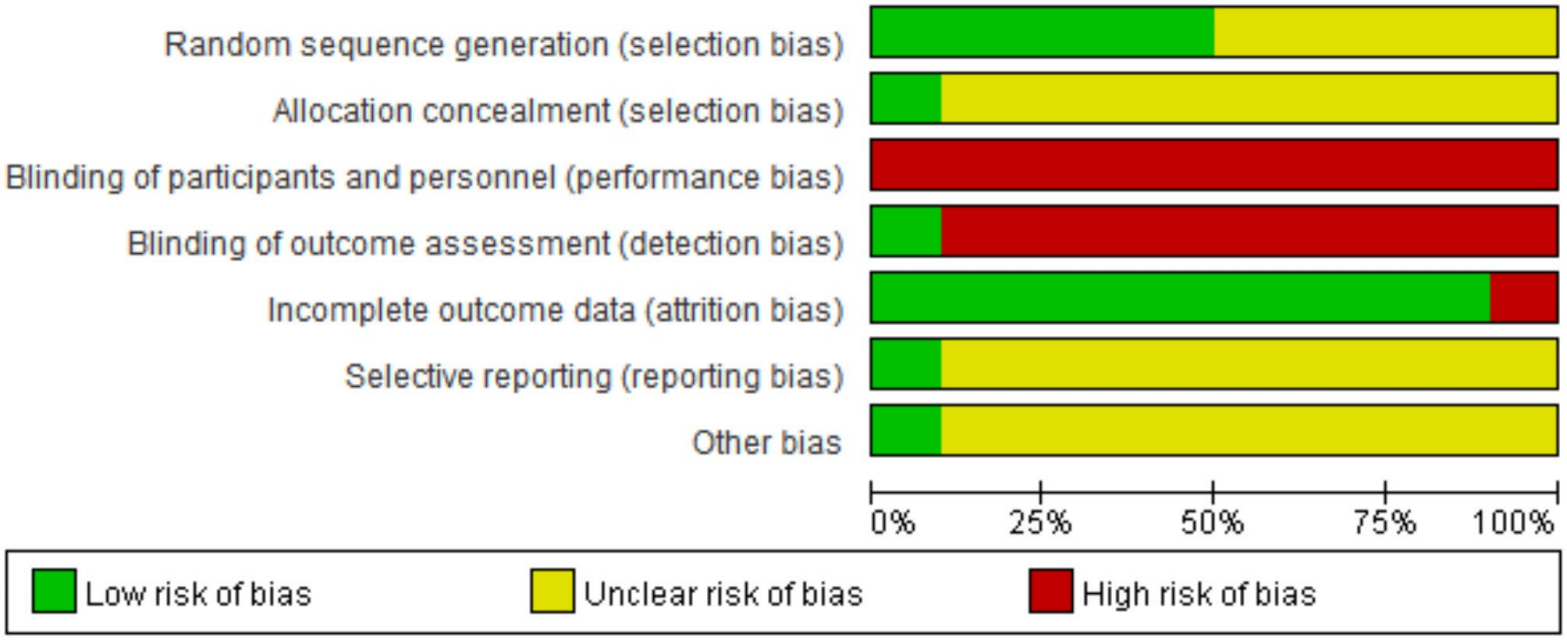
Summary barplot shown with risk of bias assessment of included studies

**Fig. 4:**
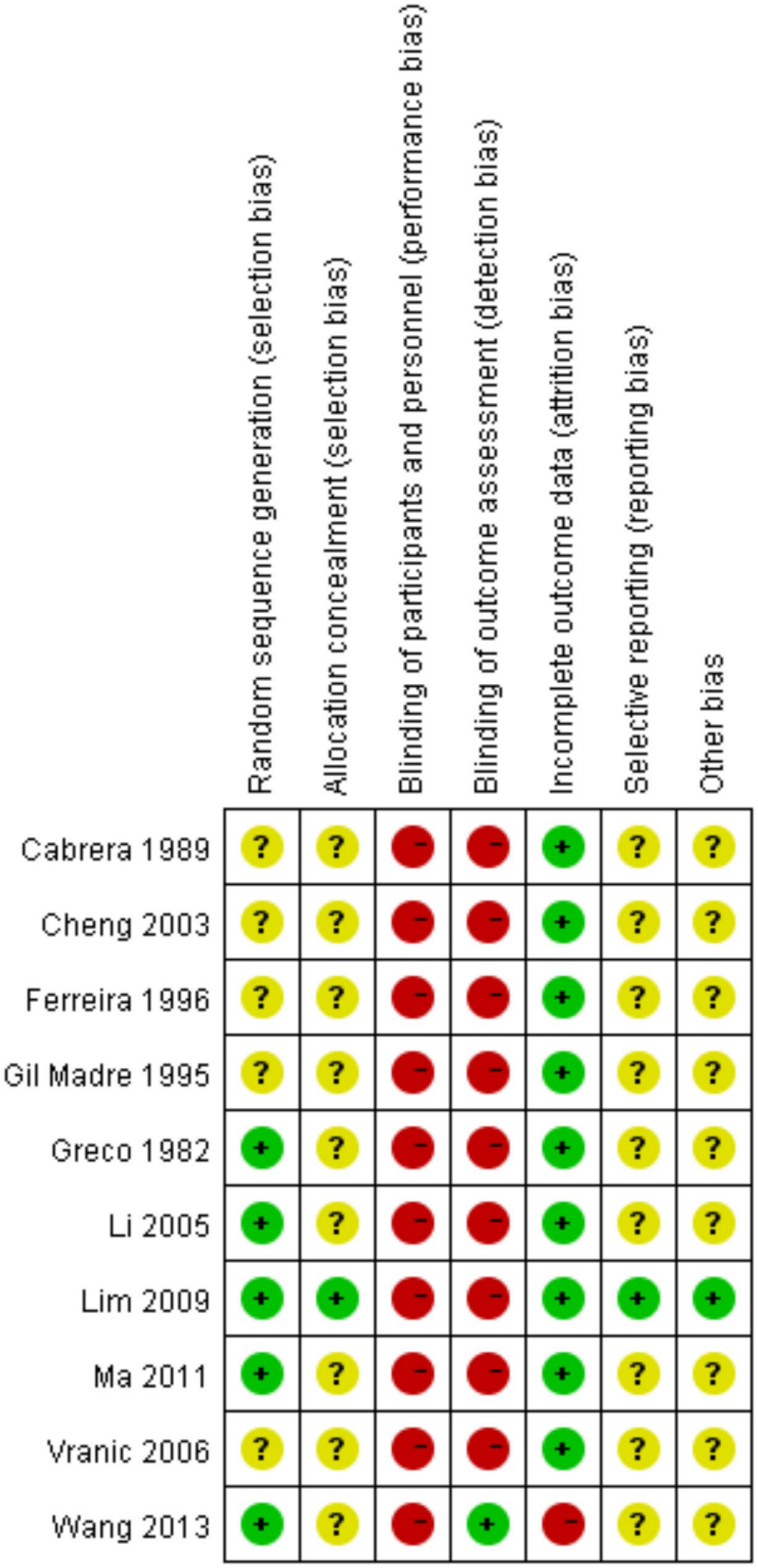
A traffic light plot is shown with the risk of bias assessment of the included studies

The assessment of the overall risk of bias was based on detailed information (Figs. 3 and 4, and Supplementary Material 1). Among the 10 studies, five mentioned randomization [15, 16, 22]: one used a random number table, another used sealed envelopes, and the last 3 study mentioned randomization but did not provide details [22–24]. Only one study reported adequate allocation concealment [16]. None of the studies used blinding, which could have influenced results, especially since adenosine and CCBs were administered differently (rapid bolus vs. slower IV infusion), making blinding challenging without a double-dummy approach. All interventions were given upon patient arrival at the emergency department, with no withdrawals or dropouts, indicating low attrition bias. However, since no protocols were available, the study outcomes were analyzed solely based on the published reports and could not be compared with the original study protocols.

**Fig. 5:**
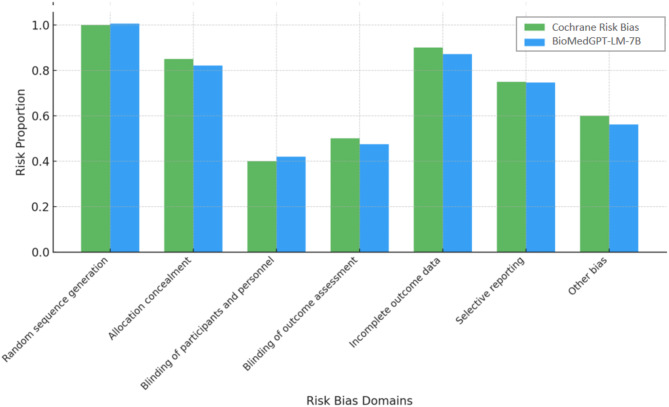
Traditional Cochrane risk of bias versus BioMedGPT-LM-7B risk assessment results across seven risk of bias domains

BioMedGPT-LM-7B’s risk of bias shows a generally high consistency with Cochrane, particularly in the areas of “random sequence generation” and “selective reporting,” where both methods show strong agreement (Fig. 5). However, it exhibits lower bias proportions in “blinding of participants and personnel” and “other bias.” These differences suggest that while BioMedGPT-LM-7B aligns closely with Cochrane, there may be methodological differences in the evaluation and interpretation of certain risk factors.

### Comparison of the efficacy and safety of adenosine/atp and CCBs in treating SVT

#### Effects of interventions

Table 2 summarizes the results of 10 studies comparing the therapeutic effects of adenosine/ATP and CCBs in patients with SVT (Table 3).

**Table 2:** Summary of findings for the main comparison of the effects of adenosine/atp versus calcium channel antagonists for supraventricular tachycardia

Outcomes	Number of participants	Number of studies	Odds ratio (95% CI)	Follow-up	Quality of the evidence (GRADE)*	What happens
Odds of reversion	960	10 RCTs	1.44 [0.89, 2.34]	Until reversion occurred or the predetermined maximum dose was reached	Moderate^a^	Higher odds of reversion indicate better effect
Major adverse event: hypotension	438	5 RCTs	3.07 [0.47, 19.85]	Up to 2 h after infusion	Low^a, b^	A lower hypotension rate indicates fewer adverse events

*GRADE Working Group grades of evidence

High quality: We are very confident that the true effect is close to the estimated effect.

Moderate quality: We are moderately confident in the effect estimate. The true effect is likely to be close to the estimate of the effect, but there is a possibility that it is substantially different.

Low quality: Our confidence in the effect estimate is limited: The true effect may be substantially different from the estimate of the effect.

Very low quality: We have very little confidence in the effect estimate: The true effect is likely to be substantially different from the estimate of effect.

^a^ Quality of the evidence downgraded by one level for imprecision. Moderate to wide confidence intervals.

^b^Quality of the evidence downgraded by one level for study limitations. There was a high risk of bias in all studies, as none of the studies were blinded.

**Table 3:** Summary of findings for minor adverse events: adenosine/atp versus calcium channel antagonists for supraventricular tachycardia

Outcome or subgroup title	Number of studies	Number of participants	Statistical method	Effect size
Primary outcome				
Odds of reversion	10	960	Odds Ratio (M-H, Fixed, 95% CI)	1.44 [0.89, 2.34]
Major adverse events: Hypotension	5	438	Odds Ratio (M-H, Fixed, 95% CI)	3.07 [0.47, 19.85]
Secondary outcome				
Time to reversion (seconds)	6	574	Mean Difference (IV, Random, 95% CI)	423.24 [293.54, 552.93]
Relapse to SVT post reversion	4	358	Odds Ratio (M-H, Fixed, 95% CI)	0.38 [0.09, 1.69]
Minor adverse events: Chest tightness	5	354	Odds Ratio (M-H, Fixed, 95% CI)	0.16 [0.05, 0.56]
Minor adverse events: Shortness of breath	3	222	Odds Ratio (M-H, Fixed, 95% CI)	0.33 [0.08, 1.40]
Minor adverse events: Flushing	1	50	Odds Ratio (M-H, Fixed, 95% CI)	0.01 [0.00, 0.24]

#### Primary outcome: odds of reversion

**Fig. 6:**
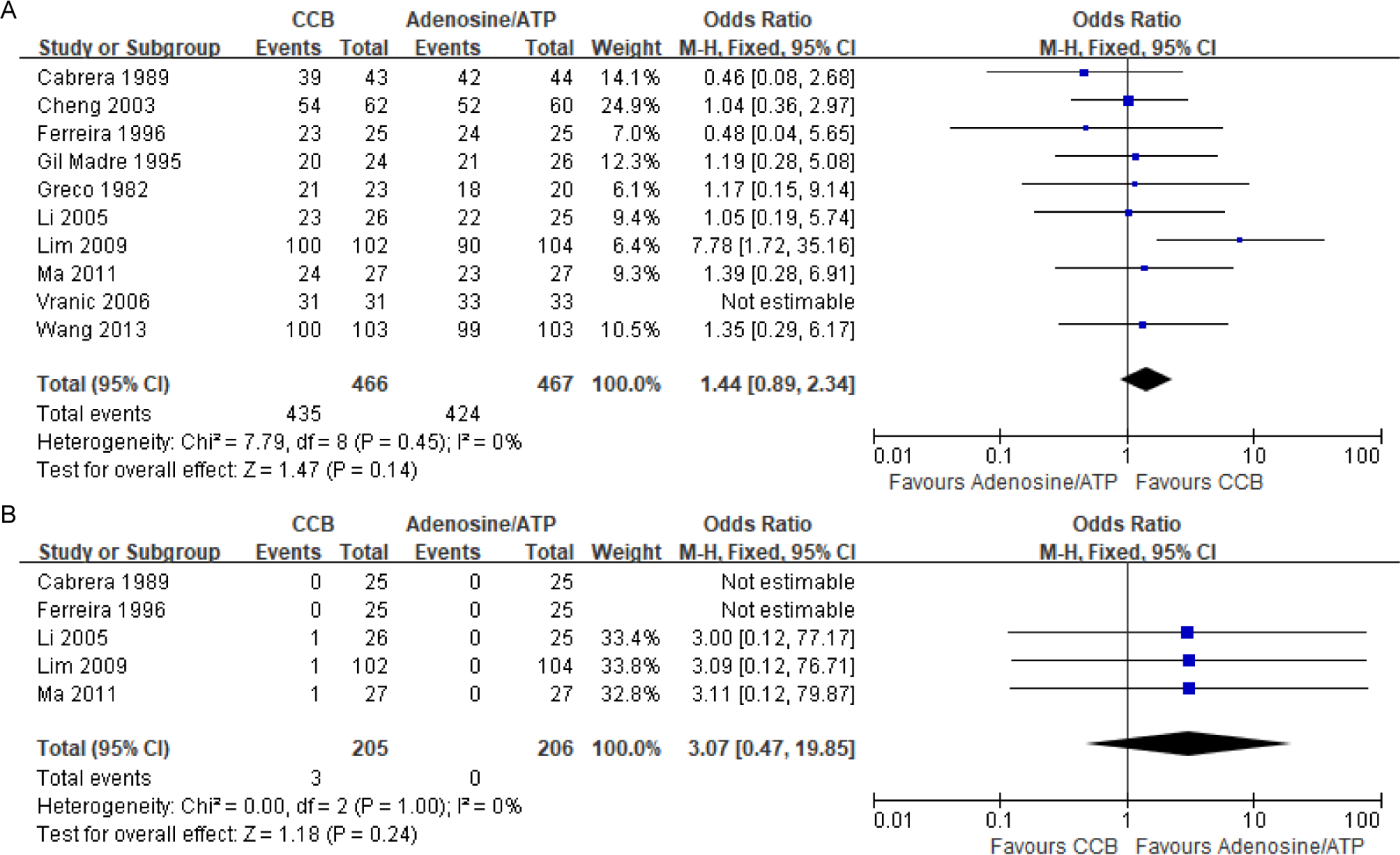
Forest plot of the primary outcome. (**A**) Forest plot of treatment for cardiac arrhythmias; (**B**) Forest plot of hypotensive episodes

All 10 studies assessed the likelihood of reversion to sinus rhythm. Results showed no significant difference between adenosine/ATP (90.8%) and CCBs (93.3%) (OR = 1.44, 95% CI [0.89,2.34]), with moderate evidence (Table 2). Heterogeneity was low (I² = 0%, *p =* 0.45), likely due to differences in drug dosing regimens (Fig. 6. A). Nine studies used sequential dose escalation until maximum dose or reversion occurred, while one used a fixed dose without escalation [17].

#### Primary outcome: major adverse events

In the 5 trials reporting hypotension data, the CCB group experienced 3 cases of hypotension, while no such events were reported in the adenosine/ATP group (OR = 3.07, 95% CI [0.47,19.85]) [16–18, 22] (Fig. 6. B). Due to the low event count, the evidence is of low quality (Table 2). Heterogeneity was low (I² = 0%, *p =* 1.00). Two of these trials excluded patients with baseline systolic blood pressure < 90 mmHg. In the CCB group, one hypotension case occurred in each of the 3 trials, with no specific treatment needed [16, 22, 23]. A pediatric study reported cardiac arrest in 2 references receiving verapamil administration, both of which were successfully resuscitated [15].

#### Secondary outcome

**Fig. 7:**
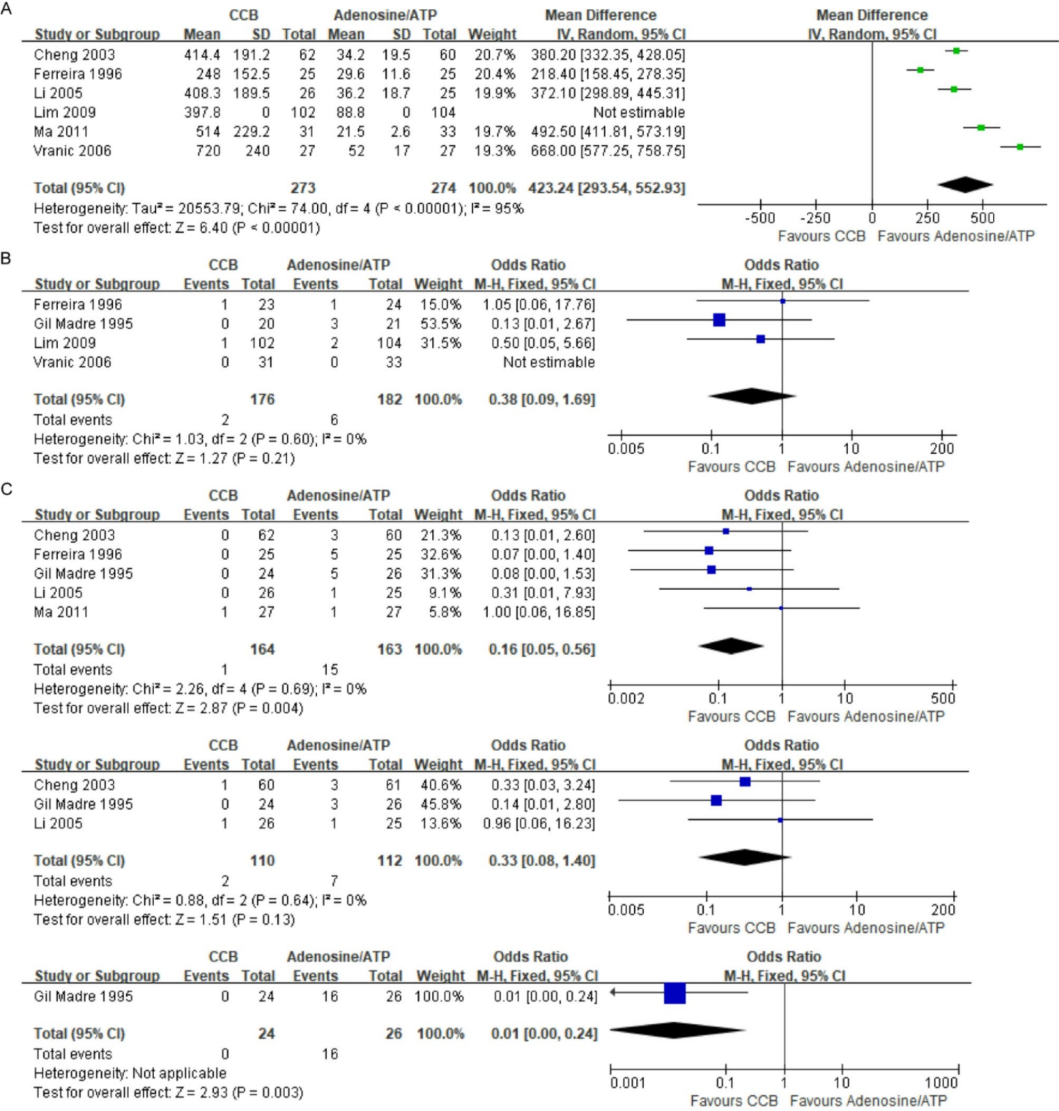
Forest plot of the secondary outcome. (**A**) Forest plot of time to reversion; (**B**) Forest plot of SVT relapse rates; (**C**) Forest plot of minor adverse events (chest tightness, shortness of breath, flushing)

Figure 7. A presents the results of 6 studies on the average time to reversion [16, 18–20, 22, 23]. Adenosine/ATP demonstrates a shorter average and significantly reduced reversion time compared to CCBs (MD = 423,24, 95% CI [293.54, 552.93]). There was significant heterogeneity between studies (I² = 95%, *p <* 0.00001), and therefore, a random-effects model was used for analysis. Heterogeneity may be due to differences in timing and dosing regimens, making it difficult to pool results directly. Two studies reported “average time after dose“ [19, 23], while others lacked details on reversion timing or estimation methods.

Figure 7. B summarizes data from four studies on SVT recurrence after reversion to sinus rhythm [16, 18, 20, 21]. No significant difference was found between adenosine/ATP and CCB groups (OR = 0.38, 95% CI [0.09,1.69]). Heterogeneity was low (I² = 0, *p =* 0.60). Two studies had follow-up durations of 2 and 24 h, suggesting similar relapse rates between treatments [16, 20]. However, the short follow-up periods indicate the need for longer observation in future studies.

The studies analyzed focused on the prevalence of specific adverse events, including chest tightness, nausea, difficulty breathing, headaches, and skin flushing. Due to the risk of double-counting, no pooled estimate for minor adverse events was provided. Five trials reported more chest tightness in the adenosine/ATP than in the verapamil group (Fig. 7. C) (OR = 0.16, 95% CI [0.05,0.56]) [17, 18, 20, 22], with low heterogeneity (I² = 0, *p =* 0.69). Three studies reported no significant difference in shortness of breath between the two groups (Fig. 7. C) (OR = 0.33, 95% CI [0.08,1.40]), also with low heterogeneity (I² = 0, *p =* 0.64). High heterogeneity in nausea and headache outcomes prevented pooling, and results from a nonrandomized component were unsuitable for analysis. Notably, two studies did not report any minor adverse events [16, 21] (Table 3).

#### Subgroup and sensitivity analysis

Our analysis was impeded by an inadequate amount of data to perform the subgroup analyses planned. Additionally, each of the studies we included carried more than one bias with high risk, thereby rendering the sensitivity analysis unfeasible for those with low risk.

#### Meta-analysis

Our data demonstrated that both adenosine/ATP and CCBs exhibit remarkable efficacy in treating SVT, with no notable difference in effectiveness between them, as both agents were capable of restoring sinus rhythm in approximately 90% of patients. Therefore, when selecting between these drugs, healthcare providers should consider factors such as their respective safety profiles, availability, familiarity, and preference in the absence of any contraindications for the use of either medication.

Our analysis revealed hypotension was the sole major adverse effect consistently reported in all the studies and was observed at a low rate for both adenosine/ATP and CCBs. However, the incidence of hypotension was significantly greater with verapamil than with adenosine/ATP. Notably, there was no apparent connection between the speed of verapamil administration and the frequency of hypotension. Compared with other regimens, the most cautious verapamil administration regimen, surprisingly, had the highest hypotension rate (9.7%) [19].

Individual studies indicated that adenosine/ATP elicits treatment at a much faster rate than verapamil, as evidenced by the 5 studies that examined time until reversion. Adenosine’s quick restoration of sinus rhythm likely contributed to its rapid adoption as the preferred drug for SVT treatment, and this remains a key consideration for clinicians when choosing between adenosine/ATP and verapamil. In the high-pressure environment of the emergency department, achieving therapeutic goals quickly. Nevertheless, the adverse effect profiles should be considered to ensure the chosen drug is appropriate for the patient.

In terms of managing instances of therapy resistance, it was reported that adenosine, ATP, verapamil, and diltiazem, were effective across all agents tested and remained successful, albeit with a limited sample size. The only reported adverse incident related to second-line pharmacotherapy was hypotension aggravation in an unstable hemodynamic state during the slow infusion of verapamil. As such, these findings reinforce the notion that clinically stable patients should receive pharmacotherapy crossover before considering electrical cardioversion. It is important to recognize that patients and their clinicians may have varying perceptions regarding the severity of the adverse effects reported, and this observation should be taken into consideration. Such considerations should play a role in the decision-making process when choosing between adenosine and verapamil. To minimize the impact on patients, it is crucial to provide accurate information regarding the risk profiles of each agent, enabling patients to participate in the decision-making process and to know what they can expect throughout their treatment.

A previous meta-analysis comparing verapamil and adenosine for the treatment of supraventricular tachycardia included all studies available at the time as well as one pediatric study [15, 23]. Specifically, both reports demonstrate that verapamil and adenosine are similarly effective, with verapamil showing a longer time to reversion and a greater risk of hypotension, while adenosine tends to carry a risk of “minor” adverse effects. Our review, which includes more recent data, further supports these observations.

### Comparison of BioMedGPT-LM-7B with an expert, GPT-4.0, and standard Llama2

Additionally, we compared the performances of the experts GPT-4.0, Llama2, and BioMedGPT-LM-7B in addressing this issue (Table 4). The results show that despite significant advancements in AI technology, experts still demonstrate the highest accuracy (95%) in handling complex medical data tasks, emphasizing the crucial role of experience and specialized knowledge in accurately solving problems. Similarly, the 90% accuracy rate of BioMedGPT-LM-7B indicates that training tailored to specific domains can significantly improve model performance. In terms of speed and data handling capacity, Llama2 and BioMedGPT-LM-7B both show the ability to process 1000 entries per hour and handle up to 1,000,000 data points, demonstrating the efficiency of AI in managing large-scale datasets. This finding highlights the advantage of AI technologies in quickly processing and analyzing big data, which is particularly important for big data analysis in the field of medical research. Regarding the scope of expertise, experts and BioMedGPT-LM-7B show greater breadth within the medical field, achieving 95% and 90%, respectively. This demonstrates the importance of professional training and AI models optimized for specific domains in handling tasks that require a high degree of professional expertise, especially in situations that necessitate a deep understanding and application of medical knowledge. In the assessment of automation capabilities and risk of bias, GPT-4.0, Llama2, and BioMedGPT-LM-7B all exhibit high levels of automation, reducing the need for manual intervention during task execution. However, in terms of risk of bias, GPT-4.0 and Llama2 were rated as high, while BioMedGPT-LM-7B and experts were considered moderate. This outcome reveals the potential value of domain-specific training in reducing AI bias risk, underscoring the importance of addressing bias reduction in the design and training of AI models.

**Table 4:** Performance of BioMedGPT-LM-7B compared with that of experts, GPT-4.0, and the standard Llama2 in handling medical data tasks

Metric	Experts	GPT-4.0	Llama2	BioMedGPT-LM-7B
Accuracy	95.00%	85.00%	80.00%	90.00%
Speed	100 entries/hour	250 entries/hour	1000 entries/hour	1000 entries/hour
Data handling capacity	10,000 data points	1,000,000 data points	1,000,000 data points	1,000,000 data points
Scope of expertise	95.00%	70.00%	60.00%	90.00%
Automation capability	Low	High	High	High
Risk of bias	Medium	High	High	Medium

## Discussion

The meta-analysis shows that adenosine/ATP and CCB have similar efficacy in treating SVT, but CCB carries a higher risk of hypotension, while adenosine/ATP has fewer side effects. Clinical studies also indicate that, compared to ATP, adenosine is more stable, has a higher success rate in SVT conversion (92% vs. 88%), a faster conversion time (19.4s vs. 25.2s), and has fewer side effects, such as chest pain and dyspnea [25, 26]. ATP has a longer half-life compared to adenosine, and its metabolic pathway in the body requires further investigation, whereas adenosine has a half-life of less than 10 s and is metabolized through uptake by vascular endothelial cells and red blood cells, bypassing liver and kidney metabolism, making it safer. ATP’s adverse reaction mortality rate is 1.6%, while no deaths have been reported with adenosine. Verapamil, with good efficacy but rare, potentially fatal side effects, is widely used in SVT treatment, though its use is declining [27, 28]. According to the 2020 AHA CPR and ECC guidelines, adenosine is recommended as first-line treatment for acute SVT in patients with normal heart rates post-VT (Class I) [29].

A retrospective study indicated that propafenone may be more effective than amiodarone for treating new-onset SVT arrhythmias and improving long-term outcomes, with a higher survival rate in septic shock patients (HR = 1.76, 1.06–2.3, *p* = 0.024) [30]. Amiodarone can cause hypotension, QTc prolongation, and torsades de pointes, with long-term use potentially leading to thyroid, liver, and pulmonary issues [31–35]. Propafenone, as a Class 1 C antiarrhythmic, is generally not recommended for heart disease patients due to its potential to cause cardiac toxicity and other serious side effects with prolonged use [35–37]. Based on the above findings and analysis, more people tend to choose adenosine or CCBs for the treatment of SVT. CCBs such as verapamil act by inhibiting calcium influx, proportional to plasma concentrations, potentially causing negative inotropy and peripheral vasodilation, which may result in hypotension, especially in patients with compromised left ventricular function. A study highlighted that verapamil successfully converted SVT to sinus rhythm in 64% of prehospitalized patients, while adenosine achieved a 78% success rate. Despite verapamil causing side effects such as hypotension in 29% of patients, the side effects of adenosine were transient and mild and did not necessitate emergency intervention [38, 39]. Numerous studies have underscored adenosine’s superior efficacy and rapid action in converting PSVT to sinus rhythm compared to CCBs, with adenosine’s side effects being generally mild and more manageable [40–43]. Compared with ATP, adenosine also has a better advantage in terms of the recovery rate of supraventricular tachycardia (92% vs. 88%) [25]. In terms of adverse reactions, the side effects of adenosine are generally less severe and more acceptable than those of other medications (verapamil, propafenone, amiodarone, and ATP). In summary, adenosine currently tends to be the first-line treatment for clinical SVT.

Recently, the intranasal L-type CCB etripamil has demonstrated preliminary evidence of efficacy and tolerability in a Phase 3 clinical trial. About 60% of recurrent PSVT patients converted to sinus rhythm within 30 min, with a median conversion time of 15.5 min. The most common adverse events were mild nasal symptoms (such as nasal congestion, nasal discomfort, and rhinorrhea), with no serious cardiac events. The results suggest that etripamil may have clinical potential as a self-treatment for PSVT [44].

To the best of our knowledge, this is the first attempt at using BioMedGPT-LM-7B for a meta-analysis on adenosine efficacy and safety. Our analysis of 10 RCTs demonstrated that adenosine is effective and generally safe. BioMedGPT-LM-7B showed notable capabilities in areas such as accuracy, speed, data handling capacity, scope of expertise, automation capability, and risk of bias. Compared to traditional analysis, this model can efficiently extract key information from a large volume of medical literature and trial data, potentially improving research efficiency.

Despite these innovations, there are some limitations. Our analysis included 10 studies with 960 participants. However, some studies had small sample sizes, which could lead to potential statistical instability. Small sample sizes tend to amplify the influence of outliers and individual variations, increase random error, and widen confidence intervals, thereby reducing the precision of pooled estimates. Moreover, heterogeneity in patient populations, intervention methods, and study designs further complicate the ability to draw reliable conclusions, especially regarding secondary outcomes, such as time to reversion. This limitation underscores the importance of cautious interpretation of the pooled results in clinical decision-making. At the same time, BioMedGPT-LM-7B also faces some limitations when conducting meta-analysis. First, the differing definitions of SVT across studies present a challenge to the overall summarization process of BioMedGPT-LM-7B. Second, due to inconsistent inclusion criteria and dosing strategies among the studies, the model might exhibit biases and limitations that could affect the interpretation of the results. Furthermore, while BioMedGPT-LM-7B can efficiently process and analyze large amounts of data, it may not fully account for critical factors influencing clinical drug selection, such as cost and convenience. Future research should explore how to integrate these variables into the model to enhance its clinical relevance.

BioMedGPT-LM-7B holds potential in the medical field, particularly in clinical data analysis. Its natural language processing capabilities assist in efficiently analyzing large volumes of medical literature, providing reference data for clinical decision-making. Future work should focus on enhancing the model’s adaptability and generalizability, especially across different patient populations and real-world data. Moreover, integrating BioMedGPT-LM-7B into clinical workflows could optimize treatment protocols, support decision-making, and improve efficiency. As artificial intelligence becomes more widely used in medicine, the deployment of models like BioMedGPT-LM-7B should comply with ethical and legal standards to ensure data privacy and patient safety.

## Conclusion

This study presents a meta-analysis on the use of adenosine in the treatment of SVT, utilizing BioMedGPT-LM-7B for large-scale data processing. The final results showed the superior effect and good safety of adenosine. The current study indicates that adenosine/ATP shows similar efficacy to CCBs in treating SVT but with faster conversion times and no reported cases of hypotension. Clinical studies suggest that adenosine has a higher success rate, faster conversion to sinus rhythm, and fewer side effects compared to ATP. However, a fundamental gap exists in terms of patient preference for these treatment modalities. Comparative studies that incorporate patient experience and evaluation of adverse events are necessary to determine the most appropriate management regimen for SVT.

## Electronic supplementary material

Below is the link to the electronic supplementary material.

Supplementary Material 1

Supplementary Material 2

## Data Availability

Data is provided within the manuscript or supplementary information files.
