# Supplementary material for: Efficacy and safety of adenosine for supraventricular tachycardia: A meta-analysis utilizing BioMedGPT-LM-7B
Source: BMC Cardiovasc Disord. 2025 Mar 7;25:158. doi: 10.1186/s12872-025-04595-x (PMC11887335; doi:10.1186/s12872-025-04595-x)
Supplement: Supplementary file 2 — Supplementary Material 2 [file 12872_2025_4595_MOESM2_ESM.docx]

**Supplementary.1 Detailed information about risk of bias.**

| **Bias - Cabrera-Sole 1989** | **Authors’ judgement** | **Support for judgement** |
| --- | --- | --- |
| Random sequence generation (selection bias) | Unclear risk | Randomisation performed, but method not specified |
| Allocation concealment (selection bias) | Unclear risk | Information insufficient to determine whether allocation concealment was adequate |
| Blinding of participants and personnel (performance bias) | High risk | Treatment was not blinded. |
| Blinding of outcome assessment (detection bias) | High risk | No attempt at blinding intervention was made. |
| Incomplete outcome data (attrition bias) | Low risk | No losses to follow-up, withdrawals, dropouts, or protocol deviations were reported. |
| Selective reporting (reporting bias) | Unclear risk | No study protocol was available for comparison of intended study outcomes vs reported outcomes. |
| Other bias | Unclear risk | No mention of funding and no mention of possible conflicts of interest |
|  |  |  |
| **Bias - Cheng 2003** | **Authors’ judgement** | **Support for judgement** |
| Random sequence generation (selection bias) | Unclear risk | Randomisation performed, but method not specified |
| Allocation concealment (selection bias) | Unclear risk | Information insufficient to determine whether allocation concealment was adequate |
| Blinding of participants and personnel (performance bias) | High risk | Treatment was not blinded. |
| Blinding of outcome assessment (detection bias) | High risk | No attempt at blinding intervention was made. |
| Incomplete outcome data (attrition bias) | Low risk | No losses to follow-up, withdrawals, dropouts, or protocol deviations were reported. |
| Selective reporting (reporting bias) | Unclear risk | No study protocol was available for comparison of intended study outcomes vs reported outcomes. |
| Other bias | Unclear risk | No mention of funding and no mention of possible conflicts of interest |
|  |  |  |
| **Bias - Ferreira 1996** | **Authors’ judgement** | **Support for judgement** |
| Random sequence generation (selection bias) | Unclear risk | Randomisation performed, but method not specified |
| Allocation concealment (selection bias) | Unclear risk | Information insufficient to determine whether allocation concealment was adequate |
| Blinding of participants and personnel (performance bias) | High risk | Treatment was not blinded. |
| Blinding of outcome assessment (detection bias) | High risk | No attempt at blinding intervention was made. |
| Incomplete outcome data (attrition bias) | Low risk | No losses to follow-up, withdrawals, dropouts, or protocol deviations were reported. |
| Selective reporting (reporting bias) | Unclear risk | No study protocol was available for comparison of intended study outcomes vs reported outcomes. |
| Other bias | Unclear risk | No mention of funding and no mention of possible conflicts of interest |
|  |  |  |
| **Bias - Gil Madre 1995** | **Authors’ judgement** | **Support for judgement** |
| Random sequence generation (selection bias) | Unclear risk | Randomisation performed, but method not specified |
| Allocation concealment (selection bias) | Unclear risk | Information insufficient to determine whether allocation concealment was adequate |
| Blinding of participants and personnel (performance bias) | High risk | Treatment was not blinded. |
| Blinding of outcome assessment (detection bias) | High risk | No attempt at blinding intervention was made. |
| Incomplete outcome data (attrition bias) | Low risk | No losses to follow-up, withdrawals, dropouts, or protocol deviations were reported. |
| Selective reporting (reporting bias) | Unclear risk | No study protocol was available for comparison of intended study outcomes vs reported outcomes. |
| Other bias | Unclear risk | No mention of funding and no mention of possible conflicts of interest |
|  |  |  |
| **Bias - Greco 1982** | **Authors’ judgement** | **Support for judgement** |
| Random sequence generation (selection bias) | Low risk | Random numbers table |
| Allocation concealment (selection bias) | Unclear risk | Information insufficient to determine whether allocation concealment was adequate |
| Blinding of participants and personnel (performance bias) | High risk | Treatment was not blinded. |
| Blinding of outcome assessment (detection bias) | High risk | No attempt at blinding intervention was made. |
| Incomplete outcome data (attrition bias) | Low risk | No losses to follow-up, withdrawals, dropouts, or protocol deviations were reported. |
| Selective reporting (reporting bias) | Unclear risk | No study protocol was available for comparison of intended study outcomes vs reported outcomes. |
| Other bias | Unclear risk | No mention of funding and no mention of possible conflicts of interest |
|  |  |  |
| **Bias - Lim 2009** | **Authors’ judgement** | **Support for judgement** |
| Random sequence generation (selection bias) | Low risk | Randomisation was performed by a nurse who drew a serialised sealed envelope. |
| Allocation concealment (selection bias) | Low risk | Participants were randomised with the use of sealed envelopes. |
| Blinding of participants and personnel (performance bias) | High risk | Interventions were given by different methods, and no attempt at blinding in tervention was made. |
| Blinding of outcome assessment (detection bias) | High risk | Not mentioned |
| Incomplete outcome data (attrition bias) | Low risk | Twenty-seven participants were excluded from analysis, as they were found not to have SVT after enrolment. Therefore, 15% of participants were not analysed in the groups to which they were randomised. However, as participants were randomised, excluded patients were closely distributed across intervention groups and had similar reasons for exclusion. |
| Selective reporting (reporting bias) | Low risk | The main outcomes reported are the same as those planned at a prospective trial registration. |
| Other bias | Low risk | Study authors declared no conflicts of interest. The Department of Clinical Re search, Singapore General Hospital, funded adenosine and diltiazem. |
|  |  |  |
| **Bias - Vranic 2006** | **Authors’ judgement** | **Support for judgement** |
| Random sequence generation (selection bias) | Unclear risk | Sequence generation and randomisation method not mentioned |
| Allocation concealment (selection bias) | Unclear risk | Not mentioned |
| Blinding of participants and personnel (performance bias) | High risk | Interventions given by different methods and no attempt at blinding intervention made |
| Blinding of outcome assessment (detection bias) | High risk | Not mentioned |
| Incomplete outcome data (attrition bias) | Low risk | Interventions were applied and outcomes were assessed within the depart ment. No losses to follow-up, withdrawals, or dropouts were reported. |
| Selective reporting (reporting bias) | Unclear risk | No study protocol was available for comparison of intended study outcomes vs reported outcomes. |
| Other bias | Unclear risk | No mention of funding and no mention of possible conflicts of interest |
| **Bias - Ma 2011** | **Authors’ judgement** | **Support for judgement** |
| Random sequence generation (selection bias) | Low risk | Randomized grouping |
| Allocation concealment (selection bias) | Unclear risk | Not mentioned |
| Blinding of participants and personnel (performance bias) | High risk | Interventions were given by different methods, and no attempt at blinding in tervention was made. |
| Blinding of outcome assessment (detection bias) | High risk | Not mentioned |
| Incomplete outcome data (attrition bias) | Low risk | Interventions were applied and outcomes were assessed within the depart ment. No losses to follow-up, withdrawals, or dropouts were reported. |
| Selective reporting (reporting bias) | Unclear risk | No study protocol was available for comparison of intended study outcomes vs reported outcomes. |
| Other bias | Unclear risk | No mention of funding and no mention of possible conflicts of interest |
| **Bias - Li 2005** | **Authors’ judgement** | **Support for judgement** |
| Random sequence generation (selection bias) | Low risk | Randomized grouping |
| Allocation concealment (selection bias) | Unclear risk | Not mentioned |
| Blinding of participants and personnel (performance bias) | High risk | Interventions were given by different methods, and no attempt at blinding in tervention was made. |
| Blinding of outcome assessment (detection bias) | High risk | Not mentioned |
| Incomplete outcome data (attrition bias) | Low risk | Interventions were applied and outcomes were assessed within the depart ment. No losses to follow-up, withdrawals, or dropouts were reported. |
| Selective reporting (reporting bias) | Unclear risk | No study protocol was available for comparison of intended study outcomes vs reported outcomes. |
| Other bias | Unclear risk | No mention of funding and no mention of possible conflicts of interest |
| **Bias - Wang 2013** | **Authors’ judgement** | **Support for judgement** |
| Random sequence generation (selection bias) | Low risk | Randomized grouping |
| Allocation concealment (selection bias) | Unclear risk | Not mentioned |
| Blinding of participants and personnel (performance bias) | High risk | Interventions were given by different methods, and no attempt at blinding in tervention was made. |
| Blinding of outcome assessment (detection bias) | High risk | Not mentioned |
| Incomplete outcome data (attrition bias) | Low risk | Interventions were applied and outcomes were assessed within the depart ment. No losses to follow-up, withdrawals, or dropouts were reported. |
| Selective reporting (reporting bias) | Unclear risk | No study protocol was available for comparison of intended study outcomes vs reported outcomes. |
| Other bias | Unclear risk | No mention of funding and no mention of possible conflicts of interest |

Abbreviation: A/E: adverse events; ATP: adenosine triphosphate; AV: atrioventricular; CCF: congestive cardiac failure; ECG: electrocardiogram; ED: emergency department; MI: myocardial infarction; RCT: randomized controlled trial; SBP: systolic blood pressure; SVT: supraventricular tachycardia; UAP: unstable angina pectoris; WPW: Wolff -Parkinson-White.
